# Ghost in the Prairie: Using Community Science and MaxEnt to Model the Habitat of a Rare Mesopredator

**DOI:** 10.1002/ece3.74140

**Published:** 2026-08-06

**Authors:** Conner A. Ties, Roel R. Lopez, Stephen L. Webb, Jessica E. Light, Benjamin W. Hoose, Ty J. Werdel

**Affiliations:** ^1^ Department of Rangeland, Wildlife and Fisheries Management Texas A&M University College Station Texas USA; ^2^ Texas A&M Natural Resources Institute Texas A&M University College Station Texas USA; ^3^ Department of Ecology and Conservation Biology Texas A&M University College Station Texas USA; ^4^ Department of Statistics Texas A&M University College Station Texas USA

**Keywords:** community science data, ecological niche modeling, habitat suitability modeling, mesocarnivore conservation, swift fox ( *Vulpes velox* )

## Abstract

Swift foxes ( *Vulpes velox* ) have experienced drastic range declines across the Southern Great Plains due to historic poisoning campaigns, land‐use change, and intraguild predation. However, recovery and conservation efforts throughout much of their range have helped mitigate or slow the effects of these threats. In Texas, however, swift fox population distributions have been reduced from 45 counties to just two. Our objectives were to identify the variables that impact potential habitat of swift fox in the Southern Great Plains and use those predictions to map potential distribution of swift fox in Texas. We used maximum entropy models (MaxEnt), community science data from iNaturalist, observer‐oriented background points, and remotely sensed data to predict potential distribution throughout the historic range of swift foxes in Texas. Highly developed cover and woody cover best predicted swift fox habitat suitability in the Southern Great Plains, both having a negative relationship with suitability. Our distribution model predicted two primary areas of potential habitat for swift foxes in Texas: one in the north‐central region of the Texas Panhandle and the other in the south‐central region of the panhandle. However, these areas are patchy with distinct clusters of greater suitability and separated by nearly 400 km, with potential barriers such as major highways and rivers impacting connectivity in the panhandle region of Texas. The model also indicates key environmental variables that are likely to contribute to suitable swift fox habitat, which can guide land management. These results can help land managers and decision‐makers identify areas of likely swift fox habitat in Texas for potential reintroductions and conservation efforts.

## Graphical abstract

Swift foxes ( *Vulpes velox* ) have experienced severe range declines in Texas, making identification of suitable habitat critical for conservation and potential reintroduction efforts. We used maximum entropy modeling with community science data from iNaturalist and remotely sensed environmental variables to predict the potential distribution of swift foxes across their historic range in Texas. Highly developed cover and woody cover were the strongest predictors of suitability, with models identifying two isolated areas of potential habitat in the Texas Panhandle that may be constrained by limited connectivity.

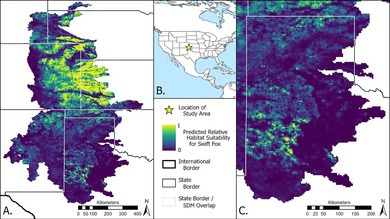

## 1 Introduction

Mammal species are facing global declines due to numerous anthropogenic threats, and carnivores are one of the most sensitive groups of mammals to human disturbance (Andermann et al. 2020; Burton et al. 2024). The loss of carnivores within their communities and landscapes leaves many important ecological roles empty, potentially having cascading effects on other taxa (Brodie et al. 2021; Burton et al. 2024). In the Great Plains region of the United States, carnivores are threatened by land‐use change (e.g., agriculture, energy production), competition and predation from invasive species, and climate change (Schmidly and Bradley 2016; Gary et al. 2019; Johnston et al. 2025). In the southern half of the Great Plains (hereafter, Southern Great Plains), these threats are exacerbated by drier and hotter conditions along with pockets of higher human population densities (Gary et al. 2019; Johnston et al. 2025). State and federal wildlife agencies in the Southern Great Plains have identified eight mammalian carnivoran species as being species of greatest conservation concern (Gary et al. 2018; Gary et al. 2019). Without management and conservation plans, these threatened carnivoran species are at risk of population decline, extirpation, or extinction due to the aforementioned threats.

The swift fox, *Vulpes velox* , is a small carnivoran (2.2–2.4 kg) that ranges throughout the arid to semi‐arid shortgrass‐dominated landscape of the Great Plains (Peek et al. 2024). Swift foxes prefer open habitats with loamy soil where they can easily detect and avoid predation, particularly from coyotes ( *Canis latrans* ; Kamler, Ballard, Fish, et al. 2003; Werdel et al. 2022; Peek et al. 2024). Swift foxes historically ranged throughout the Great Plains; however, this distribution has been reduced by nearly 50% due to land‐use change, historic carnivore poisoning campaigns, and intraguild predation by coyotes and red foxes ( *Vulpes vulpes* ; Kamler, Ballard, Fish, et al. 2003; Moehrenschlager and Sovada 2016; Thompson and Gese 2007; Werdel et al. 2022; Peek et al. 2024). The current range of the swift fox includes 10 US states (Colorado, Kansas, Montana, Nebraska, New Mexico, North Dakota, Oklahoma, South Dakota, Texas, and Wyoming) and two Canadian provinces (Alberta and Saskatchewan; Peek et al. 2024). Swift fox populations are increasing or stable in all Canadian provinces and in nine out of the 10 US states. In Texas, the statewide population is unknown and may be decreasing (Peek et al. 2024).

Monitoring and managing populations of rare species, like the swift fox, across vast heterogeneous landscapes such as the Great Plains can pose significant challenges. Increasingly, community derived data sources (e.g., iNaturalist and eBird) are being implemented to cover large heterogeneous spatial areas and generate copious amounts of data (Fontaine et al. 2022). iNaturalist alone has hosted over 316 million observations of more than half a million species as of 2026 (iNaturalist 2026). These community science datasets have become increasingly important in conservation of rare or endangered species due to their size, coverage, and accessibility (Dickinson et al. 2012; Fontaine et al. 2022). Community science has proven to be a beneficial tool to conservation by finding species that have been thought to go extinct (Losey et al. 2007), detect and monitor the spread of invasive species (Crall et al. 2015), assess how species will move across human manipulated landscapes (Werdel 2026), and directly inform conservation policy decisions (Fontaine et al. 2022). Leveraging these data sources is thus warranted for the improvement of the understanding of rare carnivores like the swift foxes and for mapping their potential habitat.

Swift foxes once ranged throughout 45 counties in Texas, primarily in portions of the High Plains and Rolling Plains ecoregions, colloquially known as the Texas panhandle (Gould et al. 1960; Schmidly and Bradley 2016). The Texas Panhandle is characterized by a mosaic of grasslands, agricultural areas, and riparian woodlands in the river valleys, with relatively few protected areas (Wiken et al. 2011). The most recently published study of swift fox occupancy in Texas (Schwalm et al. 2012) only observed them in two counties, Dallam and Sherman, in the far northwestern corner of the state, leaving 94% of the former range unoccupied.

Due to the majority of the historic range of swift foxes in Texas being unoccupied, the status of the swift fox remains uncertain in Texas (Schwalm et al. 2012). Our study aimed to (1) identify the variables that influence swift fox habitat suitability in the Southern Great Plains region, (2) leverage predictions from other states to identify potentially suitable habitat across the Texas panhandle, and (3) demonstrate that modeling with existing data can effectively inform habitat suitability while reducing the effort, cost, and logistical challenges of traditional field surveys. We predicted higher habitat suitability where intact grasslands were more prevalent, reflecting the historic association of swift foxes with open shortgrass systems.

## 2 Methods

### 2.1 Study Area

**FIGURE 1:**
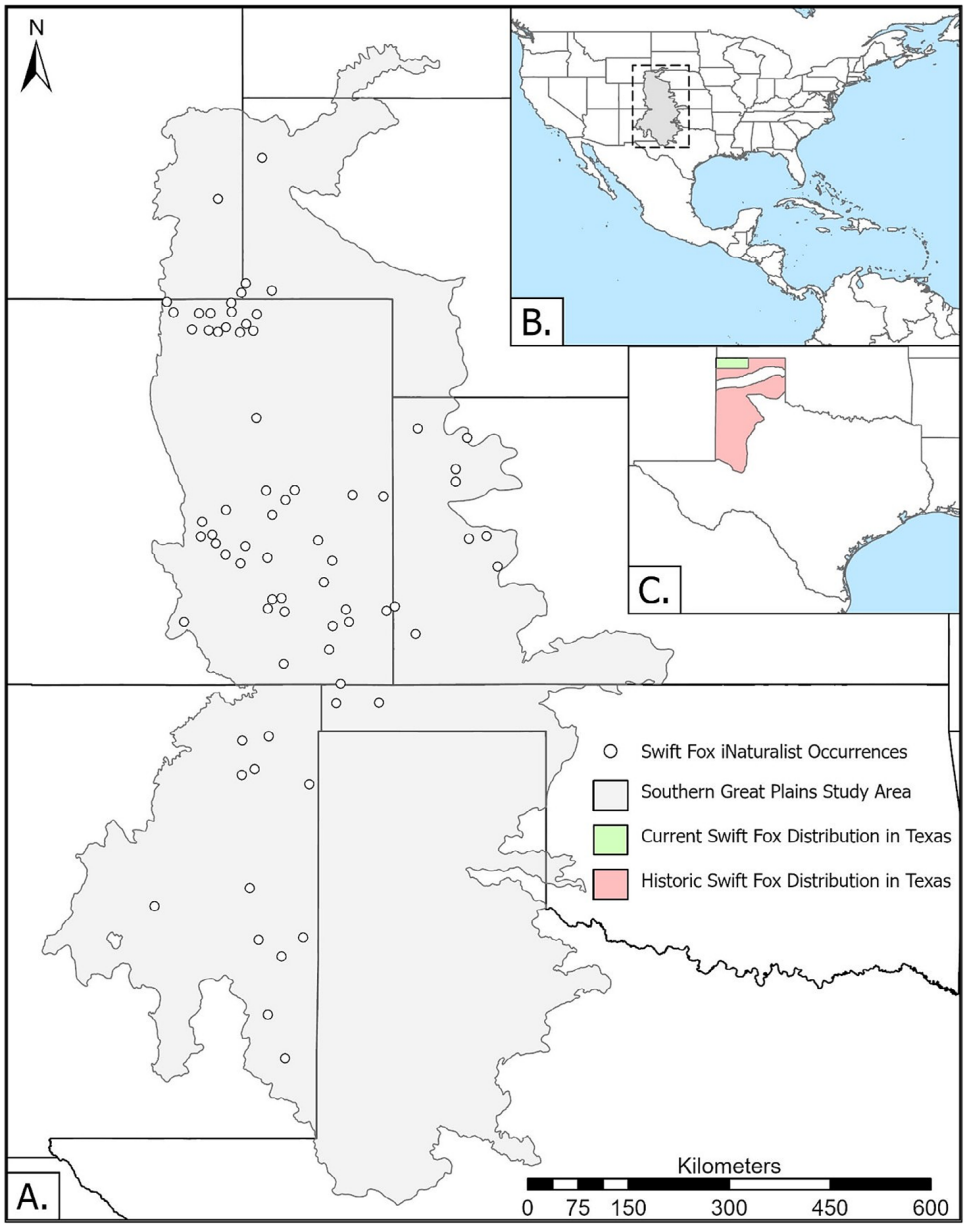
(A) Map of the southern Great Plains study area, based on EPA (2006) Level III Ecoregions, with swift fox ( *Vulpes velox* ) occurrence points from iNaturalist (2024). (B) Map of the Southern Great Plains study area location in North America. (C) Map of the historic (red) and current (green) distribution of the swift fox in Texas, United States (Moehrenschlager and Sovada 2016).

We conducted our study in the Southern Great Plains, encompassing two Environmental Protection Agency (EPA) Level III ecoregions: the High Plains and the Southwestern Tablelands, encompassing 487,149 km^2^ (Figure 1; U.S. Environmental Protection Agency 2006; Reese et al. 2016). The Southern Great Plains includes portions of Colorado, Kansas, Nebraska, New Mexico, Oklahoma, and Texas, with small portions extending into South Dakota and Wyoming (U.S. Environmental Protection Agency 2006). This region is characterized by expansive grasslands, agricultural lands, and scattered badlands (U.S. Environmental Protection Agency 2013). Average annual temperatures range from 8°C to 18°C, and average yearly precipitation ranges from 30 to 71 cm (PRISM Climate Group 2021). The predominant industries include livestock production, agricultural production (e.g., row cropping and center‐pivot irrigation), and energy production (Chapman and Bolen 2018). Gray wolves ( *Canis lupus* ) historically populated the study area; however, with their extirpation, this region has seen a nearly 50% increase in coyotes due to the lack of competition from an intraguild predator (Crabtree and Sheldon 1999; Schmidly and Bradley 2016).

### 2.2 Occurrence Data

We downloaded all research‐grade swift fox occurrence points from iNaturalist using the Global Biodiversity Information Facility (GBIF; GBIF.org 2025). Research‐grade indicates that observations were confirmed by more than two users and reached a community consensus that the species identification is correct (Van Horn et al. 2018). We filtered data using the following criteria: (1) positional accuracy, removing any occurrence point with a coordinate uncertainty greater than 1800 m based on the home range of the swift fox (Peek et al. 2024), (2) temporal extent, removing any occurrence points outside of our study time, matching the temporal range of the environmental variables used in the analysis, 2010–2024, (3) spatial extent, removing any occurrence points outside of our Southern Great Plains study area.

Due to reliance on opportunistic sampling, detection bias can be prevalent in iNaturalist data, with areas having more surveyors overrepresented in models (Supp et al. 2021). To account for this bias, we spatially thinned occurrence points using the spThin R package (version 0.2.0, R Core Team 2023; Aiello‐Lammens et al. 2015). This technique randomly removes points while enforcing a 1.8 km nearest‐neighbor distance, which is equal to the mean home range radius of a swift fox (Figure 1; Aiello‐Lammens et al. 2015; Peek et al. 2024). We used the default setting of five iterations to determine the dataset with the maximum number of points after thinning.

### 2.3 Background Data

Although randomly sampled pseudo‐absence background points are commonly used in MaxEnt modeling (Phillips et al. 2006), this approach can introduce sampling bias when working with community science data (Milanesi et al. 2020; Geurts et al. 2023). To address this concern, we implemented an observer‐oriented target‐group approach to generate background points as outlined by Milanesi et al. (2020). This method reduces sampling bias by ensuring background data reflect the same spatial coverage and observer effort as the swift fox occurrences. We used GBIF to download iNaturalist occurrences of taxa within the phylum Chordata that had an accuracy of less than 1800 m from within our study area and study period (2010–2024; GBIF 2026). We then randomly selected 10,000 background points from observers who had submitted at least one swift fox observation (Milanesi et al. 2020). This method reduces sampling bias by ensuring that background data reflect the same spatial coverage and effort as the swift fox occurrences.

### 2.4 Environmental Variables

We selected environmental variables based on the ecology of the swift fox, including woody cover, perennial forb and grass cover, annual forb and grass cover, average precipitation, clay percentage, and highly developed cover. Except for average precipitation, environmental variables were temporally matched to occurrence records (2010–2024). For average precipitation, we used 30‐year normals (1991–2020), the standard practice in species distribution modeling, to represent long‐term habitat conditions. While this period does not completely coincide with all occurrence records, there is substantial temporal overlap (2010–2020), and mean annual precipitation in the region has not exhibited major shifts in annual precipitation during this interval (Ojima et al. 2021). Therefore, we expect the impact of this temporal mismatch to be negligible. Environmental variables were either downloaded from Google Earth Engine's online public data catalog or imported into Google Earth Engine (Gorelick et al. 2017). Land cover variables (woody cover, perennial forb and grass cover, annual forb and grass cover, and highly developed cover) had a pixel size of 30 m, while clay percentage had a pixel size of 250 m. Average precipitation had a native pixel size of 928 m, which we resampled to 500 m. We chose woody cover due to its perceived negative impact on the swift fox's ability to detect predators (Kamler, Ballard, Fish, et al. 2003; Allred et al. 2021). Perennial forb and grass cover were chosen because it positively impacts swift foxes by increasing grassland habitat (Kamler, Ballard, Fish, et al. 2003; Allred et al. 2021; Werdel et al. 2023). Annual forb and grass cover have a perceived negative impact on swift foxes by decreasing native grassland habitat (Kamler, Ballard, Fish, et al. 2003; Davies 2008; Allred et al. 2021; Werdel et al. 2023). Average precipitation was included as a proxy for local environmental conditions potentially influencing habitat structure (PRISM Climate Group 2021). Clay soil percentage was chosen due to its potential negative impact on swift foxes. Greater percentages of clay content in the soil may inhibit the ability of the swift fox to dig and create burrows and this species is more likely to occur on loamy soil tablelands (Werdel et al. 2023). Lastly, highly developed cover (major roads, towns, and cities) has a perceived negative impact on swift foxes as these areas may increase human‐induced stressors (e.g., vehicle collisions, rodent control programs, secondary poisoning, and trapping pressure; Kamler, Ballard, Fish, et al. 2003; Kamler, Ballard, Gilliland, et al. 2003; USDA National Agricultural Statistics Service 2024).

### 2.5 MaxEnt Modeling

We used MaxEnt version 3.4.4 (Phillips et al. 2016), an ecological niche modeling software, to predict the relative likelihood of swift fox habitat. MaxEnt is known for its high predictive power in extrapolating presence‐only data, making it well‐suited for unstructured datasets that lack actual absence points, like iNaturalist (Elith et al. 2006; Park et al. 2006; Supp et al. 2021).

To prepare the data for MaxEnt, we imported our environmental variables, occurrence points, and observer‐oriented background points into Google Earth Engine (Figure 1; Phillips et al. 2006; Gorelick et al. 2017). We integrated occurrence and background points with environmental variables by creating a 1800 m radius circular buffer around each occurrence and background point. The buffer corresponded to an area of 10.18 km^2^ based on average home range size estimates for the swift fox (Baici and Bowman 2023; Peek et al. 2024). Within each buffer, we calculated the 30‐year annual average precipitation and clay content percentage, along with the percentage of land cover type (woody, annual forb and grass, perennial forb and grass, and highly developed). All variables showed weak or no correlation between each other (between −0.310 and 0.324), except perennial forb and grass cover and highly developed cover, which had a moderate correlation of −0.516. Thus, no variables were removed from the modeling process.

Following data preparation in Google Earth Engine, we exported occurrence and background points, along with their associated environmental variables, to the R programming language (R Core Team 2023) and standardized them to have a mean of 0 and a standard deviation of 1, given the different units used (i.e., percentages and centimeters).

Ecological niche modeling was conducted using MaxEnt through the *dismo* package (version 1.3‐14; Hijmans et al. 2023) in R. We specified a maximum of 500 iterations and used the following model parameters: 64 presence points, 10,000 background points, and all environmental variables. The MaxEnt feature types included hinge, linear, and quadratic, with regularization values of 0.153 for linear/quadratic/product, 0.250 for categorical, 1.360 for threshold, and 0.500 for hinge. Unless otherwise noted, all settings followed MaxEnt software defaults (Phillips et al. 2006).

To validate the model, we assessed its predictive performance using k‐fold cross‐validation (*k* = 4), where the occurrence and background records (with variables) were partitioned into four folds (Boyce et al. 2002; Radosavljevic and Anderson 2014). In each iteration of the cross‐validation process, we set aside one fold for testing, while the other three folds were used to train the model (Boyce et al. 2002; Radosavljevic and Anderson 2014). We generated a testing area under the receiver operating characteristic curve (AUC) from this analysis. However, as the AUC in presence‐background models such as MaxEnt evaluates discrimination between occurrence and randomly chosen background points rather than true absences, its interpretation is limited (Lobo et al. 2008; Jiménez‐Valverde 2012; Phillips et al. 2006).

Therefore, we also employed the continuous Boyce index to interpret model performance (Hirzel et al. 2006). Additionally, we calculated the mobility‐oriented parity (MOP) for the entire study area to evaluate extrapolation (Owens et al. 2013). Specifically, we computed the Euclidean distance between the values of the predictors in each pixel of the study area and the nearest 10% of values in the training data.

MaxEnt predictions were used to conduct individual variable analysis to create individual conditional expectation (ICE) plots to display the predicted relative habitat suitability for swift fox as the focal predictor varies within its observed range while all other predictors are held constant at observed values from a randomly sampled point in the study area. Along with displaying the partial dependence curves, or the average predicted relative habitat suitability across all ICE curves. ICE plots are useful for making explanatory inferences from highly flexible predictive models, such as Maxent, because they display any potential variation in the effects of predictors across the observed environmental space (Zhao and Hastie 2021).

Percent contribution (how each environmental variable contributes to the training grain of the model), permutation importance (the resulting decrease in AUC when a variable is randomly permuted), and with a jackknife (determines how regularized training gain was influenced with the removal of each variable and with just the variables) were used to investigate the importance of environmental variables in model creation (Philips 2017). We used the MaxEnt habitat suitability value predictions to develop a relative likelihood of habitat index, which was employed to map suitability across the Southern Great Plains study area in ArcGIS Pro, encompassing the current and historic range of the swift fox in Texas (Figure 1; ESRI 2011).

## 3 Results

We collected 115 swift fox occurrence points from iNaturalist across the Southern Great Plains from 2010 to 2024; none of the observations were from Texas. Following spatial thinning and removing any below a positional accuracy of 1800 m, we were left with 62 swift fox observations to be used in the model as presence points (Figure 1). For background points we collected 30,554 observations of non‐aquatic Chordata taxa from the 96 observers who had also observed a swift fox from within the study area between 2010 and 2024. Following the removal of any point below a positional accuracy of 1800 m, 18,782 observations from which the 10,000 background points were selected randomly.

**FIGURE 2:**
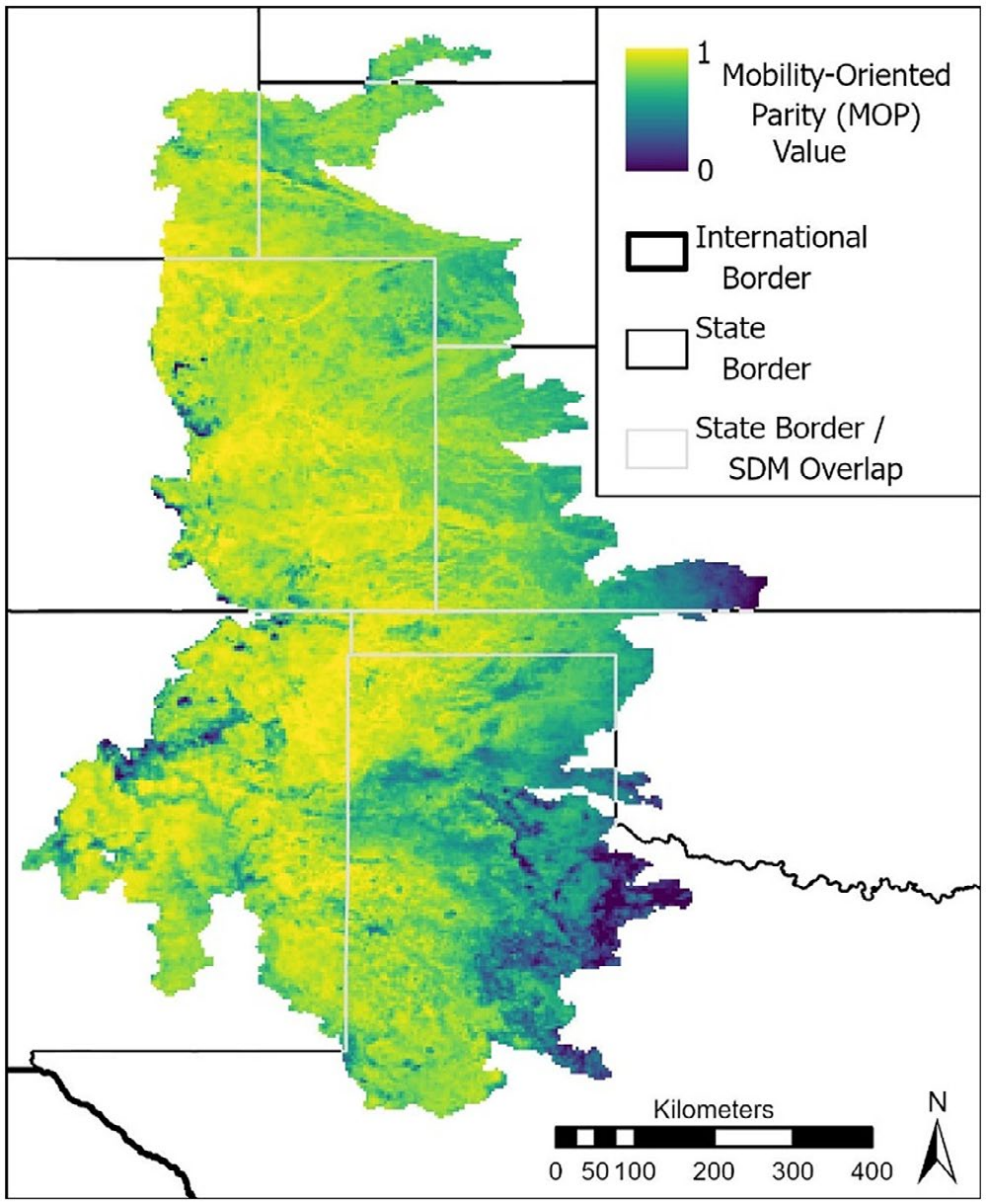
Map of mobility‐oriented parity (MOP) values for the projected swift fox habitat suitability values across the Southern Great Plains, USA (Owens et al. 2013). Values identify areas of high extrapolation (< 0.7) and low extrapolation (≥ 0.7). By computing the Euclidean distance between values of the predictors in each pixel of the study area and the nearest 10% of values in the training data.

Our MaxEnt model had a mean test AUC of 0.905 (standard deviation 0.017). While these values indicate that the model distinguishes occurrence from background points better than random, AUC in presence‐background modeling may be influenced by factors such as study extent and background selection and thus does not indicate ecological realism or predictive usefulness on its own (e.g., Lobo et al. 2008; Jiménez‐Valverde 2012). To complement this metric, we assessed model performance using the continuous Boyce index, which provides a more appropriate evaluation for presence‐only models. The model yielded an average continuous Boyce index value of 0.812 (standard deviation 0.083). Both validation approaches indicate the model discriminates presence from background records, but biological inference should rely primarily on the Boyce index. Mean mobility‐oriented parity (MOP) value for the model was 0.773 indicating low extrapolation for the entirety of the Southern Great Plains. However, areas of lower MOP values (higher extrapolation) were found on some edges of the study area, a branch of the Southern Great Plains into southern Kansas, and the eastern Texas Panhandle (Figure 2). But the mean MOP value for the Texas Panhandle was 0.717 and over 63.7% of the region had a greater MOP value than 0.7.

**FIGURE 3:**
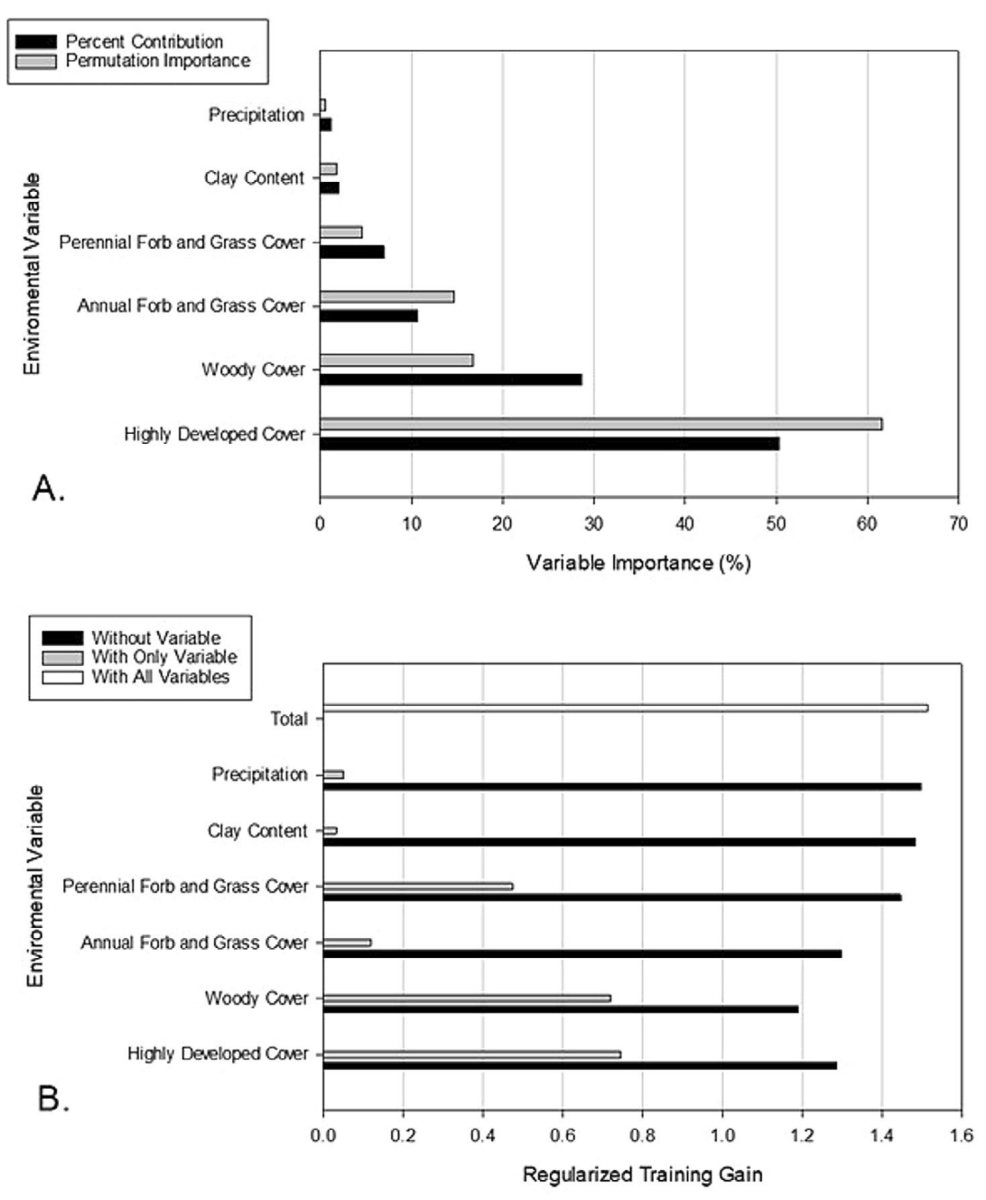
(A) Percent contribution and permutation importance of environmental variables used in the MaxEnt model for swift fox ( *Vulpes velox* ) habitat suitability across the Southern Great Plains, United States. Bars represent the relative influence of each environmental covariate in model training, with percent contribution (black) indicating each variable's additive effect on model gain, and permutation importance (gray) representing the decrease in model performance (AUC) when variable values are randomly permuted. (B) Jackknife test of variable importance in the MaxEnt model for swift fox habitat suitability across the Southern Great Plains, US. The white bar represents the total regularized training gain of all variables. Gray bars represent regularized training gain with only that variable. Black bars represent regularized training gain without the variable.

**FIGURE 4:**
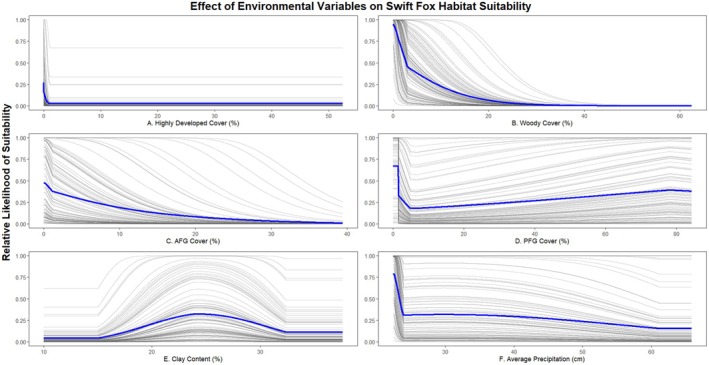
Individual conditional expectation (ICE) plots for each predictor (A) Highly Developed Cover, (B) Woody Cover, (C) Annual Forb and Grass Cover, (D) Perennial Forb & Grass Cover, (E) Clay Content, and (F) Average Precipitation in the ecological niche model. The light gray lines display the predicted relative habitat suitability for swift fox as the focal predictor varies within its observed range while all other predictors are held constant at observed values from a randomly sampled point in the study area. Blue lines display the partial dependence curves, or the average predicted relative habitat suitability across all ICE curves. ICE plots are useful for making explanatory inferences from highly flexible predictive models, such as Maxent, because they display any potential variation in the effects of predictors across the observed environmental space (Zhao and Hastie 2021).

Variables that contributed most to model creation/training and environmental variables importance were highly developed cover (percent contribution (PC) = 50.3%; permutation importance (PI) = 61.6%), woody cover (PC = 28.7%; PI = 16.8%), and annual forb and grass cover (PC = 10.7%; PI = 14.7%; Figure 3A). While perennial forb and grass cover (PC = 7%; PI = 4.6%) contributed to a lesser degree, clay content (PC = 2%; PI = 1.8%), and precipitation (PC = 1.2%; PI = 0.6%) had less contribution to model creation/training and environmental variable importance (Figure 3A). Jackknife results showed that highly developed cover had the highest gain when used in isolation, followed by woody cover (Figure 3B). While removing woody cover decreased the gain the most, this indicated that woody cover had the most information not found in other variables. The three most impactful variables in the model—highly developed cover, woody cover, and annual forb and grass cover—exhibited a negative relationship with habitat suitability (Figure 4A–C). Any percentage of highly developed cover was associated with low likelihood of suitability (Figure 4A). Habitat suitability decreased quickly as woody cover increased from 0% to 10% (Figure 4B). Increases in annual forb and grass cover displayed a decrease in habitat suitability, which remained constant or slightly decreased with increasing annual forb and grass cover (Figure 4C). Perennial forb and grass cover showed a steep decline between 0% and 5%, with habitat suitability increasing with perennial forb and grass cover beyond 5% (Figure 4D). Habitat suitability was greater where clay content ranged between 20% and 30%, compared to areas with clay content outside this range (Figure 4E). As average precipitation increased, habitat suitability decreased (Figure 4F).

**FIGURE 5:**
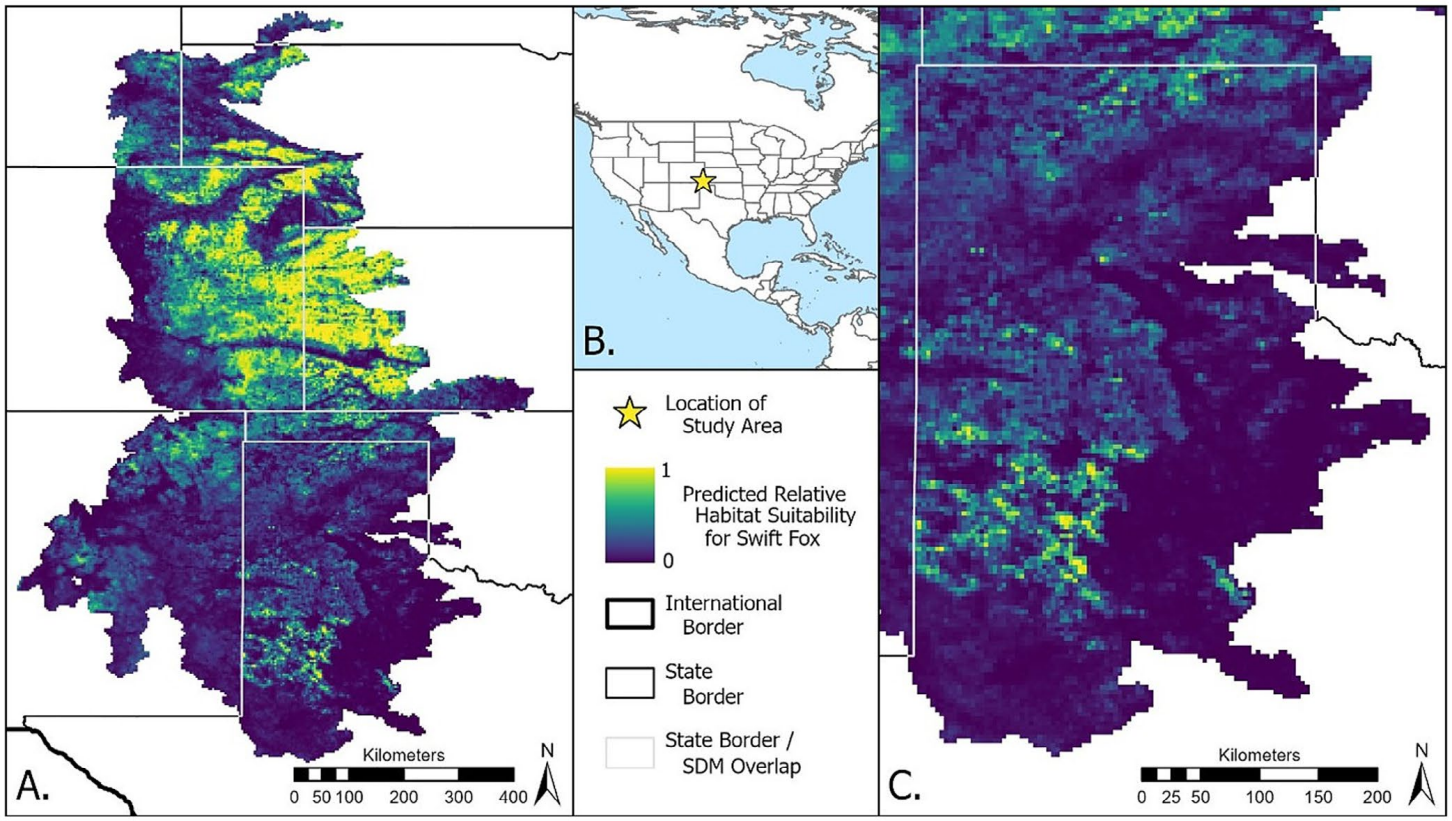
(A) Relative likelihood of habitat suitability of swift fox ( *Vulpes velox* ) in the Southern Great Plains, US. Based on MaxEnt results using community science data. Values ranged from 0 (low) to 1 (high) likelihood of habitat suitability. (B) Location of the study area (yellow star) within North America. (C) Relative likelihood of habitat suitability of swift fox ( *Vulpes velox* ) in the Texas Panhandle, US. Based on MaxEnt results using community science data. Values ranged from 0 (low) to 1 (high) likelihood of habitat suitability.

Predicted habitat suitability ranged from 0 to 1 across the Southern Great Plains and the Texas Panhandle (Figure 5A,C). The mean pixel value was 0.271 for the entire Southern Great Plains and 0.123 for the Texas Panhandle. Suitable habitat greater than 0.50 habitat suitability was found throughout the Southern Great Plains; however, the largest patches were found in the north central portion of the study area in eastern Colorado, western Kansas, and southwestern Nebraska (Figure 5A). In the Texas Panhandle, areas with greater suitability were found in the northeast‐central and southwest‐central areas of this region (Figure 5C).

## 4 Discussion

These results demonstrate that, when carefully filtered and analyzed to address inherent bias, community science data can provide valuable information on rare data‐deficient mesocarnivores. Generated distribution maps could help improve management and conservation. While we predicted that variables contributing to intact open grasslands would be the leading factors in swift fox suitability model, highly developed cover was the most impactful followed by woody cover indicating that open habitat was important to the model (Figure 3). These results demonstrate that, when carefully filtered and analyzed to address inherent bias, community science data can provide valuable insights (Aiello‐Lammens et al. 2015).

Our model found that highly developed cover was the strongest predictor of habitat suitability, followed closely by woody cover. Both variables show a negative relationship with suitability. These findings indicate that swift foxes prefer vast, open areas with minimal human development and tree/shrub‐free landscapes, and align with ecological patterns reported in previous studies, as roads were identified as the primary cause of swift fox mortality (Kamler, Ballard, Fish, et al. 2003). The avoidance of areas with higher woody cover may be explained, at least in part, by reduced visibility and a diminished ability to detect predators such as coyotes (Kamler, Ballard, Gilliland, et al. 2003; Thompson and Gese 2007; Werdel et al. 2022).

We collected 115 swift fox observations from iNaturalist (the only community science database used) across the Southern Great Plains over a 14‐year period. Notably, there were no iNaturalist observations from Texas; however, there were observations from the surrounding states of New Mexico and Oklahoma (Figure 1). While this absence could indicate habitat loss or reduced populations, it may also reflect limited reporting rather than a true absence. We identified disjunct, localized areas of more suitable habitat, with implications for population persistence.

Greater swift fox habitat suitability was found throughout the Southern Great Plains study area (Figure 5A). Most large contiguous patches were found in the northern half of the area, while the southern half (made up by a majority of the Texas Panhandle) had smaller scattered patches indicating less contiguous habitat (Figure 5A). Texas has seen a century of land cover change and rapid urban development, potentially causing the decrease in the geographic range of swift foxes in the state. Historically, the swift fox ranged throughout 45 counties in Texas, including most of the Texas Panhandle (Figure 1C; Schmidly and Bradley 2016). However, Schwalm et al. (2012) found swift foxes in only two counties, Dallam and Sherman Counties, located in the far northwestern panhandle. Our model suggests suitable habitats in Texas still occur in the north‐central and south‐central areas of the panhandle region of Texas, with significant gaps between them, indicating potential barriers to connectivity. However, suitable habitat does not necessarily indicate that populations exist within these suitable patches. Dispersal between the two larger patches of suitable habitat in Texas is likely limited by multiple barriers, including the Canadian River valley, smaller river valleys, and several major highways (Kamler, Ballard, Fish, et al. 2003). The north‐central area is also more connected to suitable habitat in the neighboring state of Oklahoma, while the south‐central area is not connected to any suitable habitat area in other states.

For swift fox populations to persist or recover in Texas, it will be necessary to conserve remaining suitable habitat and implement management strategies that reduce woody encroachment across the landscape, specifically keeping woody cover below 5% on the landscape. Additionally, further survey efforts should be conducted to confirm or deny the presence of swift foxes in the state of Texas, as the last recorded sighting in the literature dates back more than a decade (Schwalm et al. 2012). Additionally, because Texas is a majority private‐land state and most suitable habitat occurs on private land, effective conservation efforts must prioritize engaging private landowners (Texas A&M Natural Resources Institute [NRI] 2025).

Understanding habitat suitability for a given species is crucial for future conservation planning (Elith et al. 2006). Our swift fox model and prediction map can play an essential role in the future of this species in Texas. By determining the relationships between environmental variables and swift fox habitat suitability, land managers can enhance swift fox habitat by removing woody cover and maintaining an open landscape. Additionally, the results of this study show that combining MaxEnt with community science data can provide valuable insights for conservation, especially when resources are limited. This approach offers a cost‐effective means to inform management decisions, map potential habitat, and guide future field efforts for swift fox and other species of conservation concern.

## Author Contributions

**Conner A. Ties:** conceptualization (lead), data curation (lead), formal analysis (equal), investigation (lead), methodology (lead), software (equal), validation (lead), visualization (lead), writing – original draft (lead), writing – review and editing (lead). **Roel R. Lopez:** conceptualization (supporting), funding acquisition (supporting), writing – review and editing (supporting). **Stephen L. Webb:** writing – review and editing (supporting). **Jessica E. Light:** writing – review and editing (supporting). **Benjamin W. Hoose:** conceptualization (supporting), data curation (supporting), formal analysis (equal), investigation (supporting), methodology (supporting), software (equal), validation (supporting), writing – review and editing (supporting). **Ty J. Werdel:** conceptualization (supporting), funding acquisition (lead), investigation (supporting), methodology (supporting), supervision (lead), validation (equal), writing – review and editing (supporting).

## Funding

This work was supported by the Houston Safari Club Foundation, Dan L. Duncan Scholarship.

## Conflicts of Interest

The authors declare no conflicts of interest.

## Data Availability

Data and code are openly available on Dryad at https://doi.org/10.5061/dryad.j0zpc86x0.
